# Leishmaniasis in Central Morocco: Seasonal Fluctuations of Phlebotomine Sand Fly in Aichoun Locality, from Sefrou Province

**DOI:** 10.1155/2015/438749

**Published:** 2015-02-08

**Authors:** Fatima Zahra Talbi, Abdelhakim El Ouali Lalami, Abdellatif Janati Idrissi, Faiza Sebti, Chafika Faraj

**Affiliations:** ^1^Analysis and Modelisation of Continental Ecosystems Laboratory, Faculty of Sciences Dhar El Mehraz, Sidi Mohamed Ben Abdellah University, 30000 Fes, Morocco; ^2^Laboratory of Medical Entomology, National Institute of Hygiene, 27 Avenue Ibn Battuta, Agdal, 11400 Rabat, Morocco; ^3^Regional Diagnostic Laboratory Epidemiological and Environmental Health, Regional Health Directorate, EL Ghassani Hospital, 30000 Fes, Morocco; ^4^National Reference Laboratory of Leishmaniasis, National Institute of Hygiene, 27 Avenue Ibn Battuta, Agdal, 11400 Rabat, Morocco

## Abstract

Cutaneous leishmaniases (CL) are endemic in Morocco. They are common in the human population in different localities such as Aichoun in Sefrou province, Morocco. This study was carried out in Aichoun locality from April to October 2012 in order to study the spatiotemporal trends of the main *Leishmania* phlebotomine vectors in this focus. Overall, 1171 sand flies, belonging to four species, were collected by sticky traps. *Phlebotomus sergenti* was the predominant species (78.4%) followed by *Ph. perniciosus* (10.5%), *Ph. papatasi* (7.94%), and *Ph. longicuspis* (3.16%). Sandflies were active during 6 months (May–October). *Ph. sergenti, Ph. perniciosus*, and *Ph. papatasi* displayed a bimodal distribution with a first peak in July and a second peak in September, while *Ph. longicuspis* showed a monophasic trend with a peak in August. The high abundance and the lengthy period of activity of *Ph. sergenti* and *Ph. perniciosus*, vectors of *L. tropica* and *L. infantum*, respectively, are a cause for concern as they indicate the high potential risk of *Leishmania* transmission in the studied areas.

## 1. Introduction

Cutaneous Leishmaniasis (CL) is an increasingly public health problem in Morocco. Three forms are present: anthroponotic leishmaniasis caused by* Leishmania tropica* and zoonotic leishmaniasis caused by* L. major* and less frequently by* Leishmania infantum* [1]. The parasites are transmitted by phlebotomine sand flies, namely,* Phlebotomus sergenti* for ACL and* Ph. papatasi* and phlebotomines from* Larrousius* subgenus for* L. major* and* L. infantum*, respectively, for ZCL [2]. The main reservoir host for* L. major* is considered to be the rodent* Meriones shawi* [1].

CL caused by* L. tropica* is the most widespread in semiarid areas in central and south-western Morocco. This disease was reported for the first time in Azilal province in 1989 [3], then in other focus in central and southern areas of the country such as Guelmim, Agadir, Essaouira [4], and Taza [5]. CL due to* L. infantum* was reported in Taounate province in 1996, within an active focus of visceral leishmaniasis [6].* L. infantum* is also responsible for widespread visceral leishmaniasis in the north-eastern slope of the Rif Mountains when dog is the main domestic reservoir of the parasite [7].

Each year, we witness the emergence of new foci, extending the disease distribution and increasing its total impact. In 2011 and 2012, the Ministry of Health recorded, respectively, 4426 and 2990 cases with 4.92% and 7.19% in the region of Fes-Boulmane [8, 9]. The region of Fes-Boulmane recorded the first outbreak of CL in 2001 with 1600 cases in the province of Moulay Yacoub alone [10, 11]. The outbreak was rapidly stopped by insecticides use, but the disease has persisted since that under endemic and epidemic forms in other provinces of the region with more than 100 cases each year despite a strong monitoring plan.

The control of the disease is mainly based on case detection and treatment of human cases and on vector control. This is based essentially on environmental management including promotion of improved solid waste disposal practices. The use of bed nets and local residual indoor spraying with synthetic pyrethroids are often applied in newly emerged epidemic foci. Nevertheless, few data are available on the vector* Ph. sergenti*, especially seasonal fluctuations of its abundance. This information is essential for the success of vector control. In this context, we have conducted this study for the first time in a village belonging to the region of Sefrou province in center of Morocco.

This study also allows a better understanding of the dynamics of the transmission of leishmaniasis in the area of Aichoun and, therefore, contributes to the future design of surveillance strategies.

## 2. Materials and Methods

### 2.1. Study Area and Environment

The entomological study was conducted in the locality of Aichoun (33°39′N, 04°38′W) situated in the northwest of the Moroccan Middle Atlas, a part of the province of Sefrou that belongs to the territory pastoral village of Tazouta with a cold semiarid climate. The mean altitude is 750 m. The average annual rainfall is 400 mm, the main minimum of the coldest month is 2°C, and maximum of the hottest month is 40°C.

The locality of Aichoun is characterized by the presence of cowsheds, caves, and accumulation of animal waste that creates an environment favorable to the biological cycle of sand flies. The vegetation is characterized by a covering more or less degraded because of overgrazing and overexploitation for domestic uses (*Tetraclinis articulata, Pistacia lentiscus, Olea oleaster, Olea europaea,* and* Juniperus phoenicea*).

### 2.2. Sand Fly Collection and Identification

Sand flies were collected bimonthly during their optimum development period in Morocco, between May and October. Five collecting sites were chosen in Aichoun locality. Collections were performed by using sticky papers (21 × 27,3 cm) coated with castor oil. We used 40 traps in each trapping campaign. The traps were installed in different habitats during the day and were removed the next day. Collected sand flies were transferred in glass tubes containing a solution of ethanol at 70° . After sex determination, all sand flies collected were identified by examining the morphology of the pharyngeal armature and spermathecae of female flies and the external genitalia of males using the morphological key [12, 13]. Morphological differentiation of the two sympatric species* Ph. longicuspis* and* Ph. perniciosus* was made according to description of Berchi et al. [14].

## 3. Results

### 3.1. Taxonomic Inventory of Sand Flies

This study has established a faunal inventory of Aichoun revealing the presence of four species belonging to the genus* Phlebotomus.* The sex ratio indicated that more males were collected; the male/female ratio was 7.54.

The collections accounted for 1171 sand fly specimens.* Ph. sergenti* was the predominant and the most frequently collected species with 918 individuals (78.4%). In second place comes* Ph. perniciosus* Newstead with 123 (10.50%) followed by* Ph. papatasi* Scopoli with 93 individuals (7.94%).* Ph. longicuspis* Nitzulescu was represented by 3.16% (Table 1).

### 3.2. Seasonal Fluctuations of Sand Flies Species

**Figure 1:**
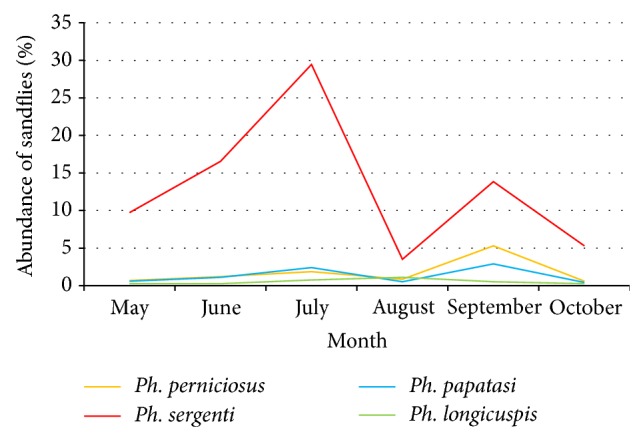
Seasonal variation of sand flies collected in Aichoun by sticky paper traps.

Seasonal activity of sand flies extended from May to the end of October, with variation in the monthly evolution according to species.* Ph. sergenti*,* Ph. Perniciosus,* and* Ph. papatasi* displayed a bimodal distribution with a first peak in July, where* Ph. sergenti* dominates (29.46%), and a second peak in September with 13.83%, while* Ph. longicuspis* showed a monophasic trend with a slight peak in August (1.11%) (Figure 1).

## 4. Discussion

Over the last years, incidence of* L. tropica* CL has continuously increased in the province of Sefrou [15]. Between 1997 and 2011, 1242 cases were declared [16]. The majority of cases were reported mainly in El Menzel, Sefrou, Aghbalou, Tazouta, Sidi Lahcen, and Ain Chegag communes [17].

*Ph. sergenti* is confirmed as vector of* L. tropica* in northern Africa, Middle East, and Central Asia [18, 19], the same in Morocco [4]. It dominates in arid and semiarid areas [20]. In this study area,* Ph. sergenti* was largely widespread; it was present throughout the whole period of our surveys, showing a biphasic course evolution with two peaks in July and September, respectively. Its activity period was investigated in Taza, a semiarid area in northern Morocco, where it was collected from June to November showing two density peaks [21].* Ph. sergenti* was the dominant species compared to other species, which confirms the results obtained in 2011 [22] and with a maximum activity in July confirming the results obtained in Marrakech province [23].

*Ph. perniciosus,* one of the most competent* L. infantum* vectors in the Mediterranean foci [24], was present during all the period of our study. This species showed a biphasic activity and dominating in September (5.29%). This is in line with the results obtained by Guernaoui et al. [25] in the province of Chichaoua, southwest of Morocco.

*Ph. papatasi* was also present in our study even if it is considered to be adapted to arid climate [26];* Ph. papatasi* populations were present from May to October with two peaks. These results are in accordance with the data obtained in Marrakech area in southwestern Morocco [27].

*Ph. longicuspis* was collected in Aichoun locality. The highest density of this species was observed in August. This result is in accordance with Guernaoui et al. [28] in Chichaoua province where this species showed a monophasic cycle, with one density peak in August-September.

In the region of Fez Boulmane one similar study of the species inventory sand flies in a focus of cutaneous leishmaniasis nominated Ouled aid and belonging to a neighboring province than that Sefrou was realized in 2011 during a year between April 2011 and March 2012 [29, 30]. If we compare our results with those found in this study, we can infer that the same species were encountered but not in the same abundance. Indeed, the most abundant species found were, respectively,* Phlebotomus papatasi* (50.52%),* Phlebotomus sergenti* (24.7%),* Phlebotomus perniciosus* (9.69%), and* Phlebotomus longicuspis* (5.54%) [29].

According Lahouiti et al. [30], the destruction of vectors' habitats, the improvement of human habitats, the treatment of scraps, animal drop-pings, dung, and the separation between animal and human dwellings could be an efficient way to get rid of sand flies and consequently diminish the risks of contamination.

In addition to means mentioned above, we must remember that the companions of information-education-communication and the process of integrated management of vector control piloted together by the health services are very efficient tools to prevent cases of leishmaniasis.

Also, these means will be very efficient not only to fight the mosquito sand fly vector of such diseases but also to combat the parasite tanks such as rats and stray dogs who are increasingly responsible for many emerging zoonoses [31, 32].

## 5. Conclusion

This study, conducted in Aichoun locality during the months of April to October 2012, was aimed at studying the spatial and temporal trends of the main vectors of* Leishmania* in sand flies in this focus. 1171 sand flies, belonging to the four species, were collected by sticky traps.* Phlebotomus sergenti* was the predominant species (78.4%) followed by* Ph. perniciosus* (10.5%),* Ph. papatasi* (7.94%), and* Ph. longicuspis* (3.16%). Sand flies were active during 6 months (May–October).

The large distribution and the long activity period of* Ph. sergenti* and species of the subgenus* Larroussius* in Aichoun locality indicate the high potential risk of* L. tropica* and* L. infantum* transmission in this area.

## Figures and Tables

**Table 1:** Sandflies collected in Aichoun locality, 2012.

Subgenus (R. Ab. %)	Species	Mal	Female	Total	R. Ab. (%)
*Larroussius* (13.74)	*Phlebotomus longicuspis *	13	24	***37***	3.16
	*Phlebotomus perniciosus *	113	10	***123***	10.50

*Paraphlebotomus* (78.28)	*Phlebotomus sergenti *	845	73	***918***	**78.4**

*Phlebotomus* (7.98)	*Phlebotomus papatasi *	63	30	***93***	7.94

R. Ab.: Relative Abundance.
